# Supplementary material for: INTACT vs. FANS for Cell-Type-Specific Nuclei Sorting: A Comprehensive Qualitative and Quantitative Comparison
Source: Int J Mol Sci. 2021 May 19;22(10):5335. doi: 10.3390/ijms22105335 (PMC8159132; doi:10.3390/ijms22105335)
Supplement: Supplementary file 1 [file ijms-22-05335-s001.zip › ijms-1154486-supplementary.pdf]

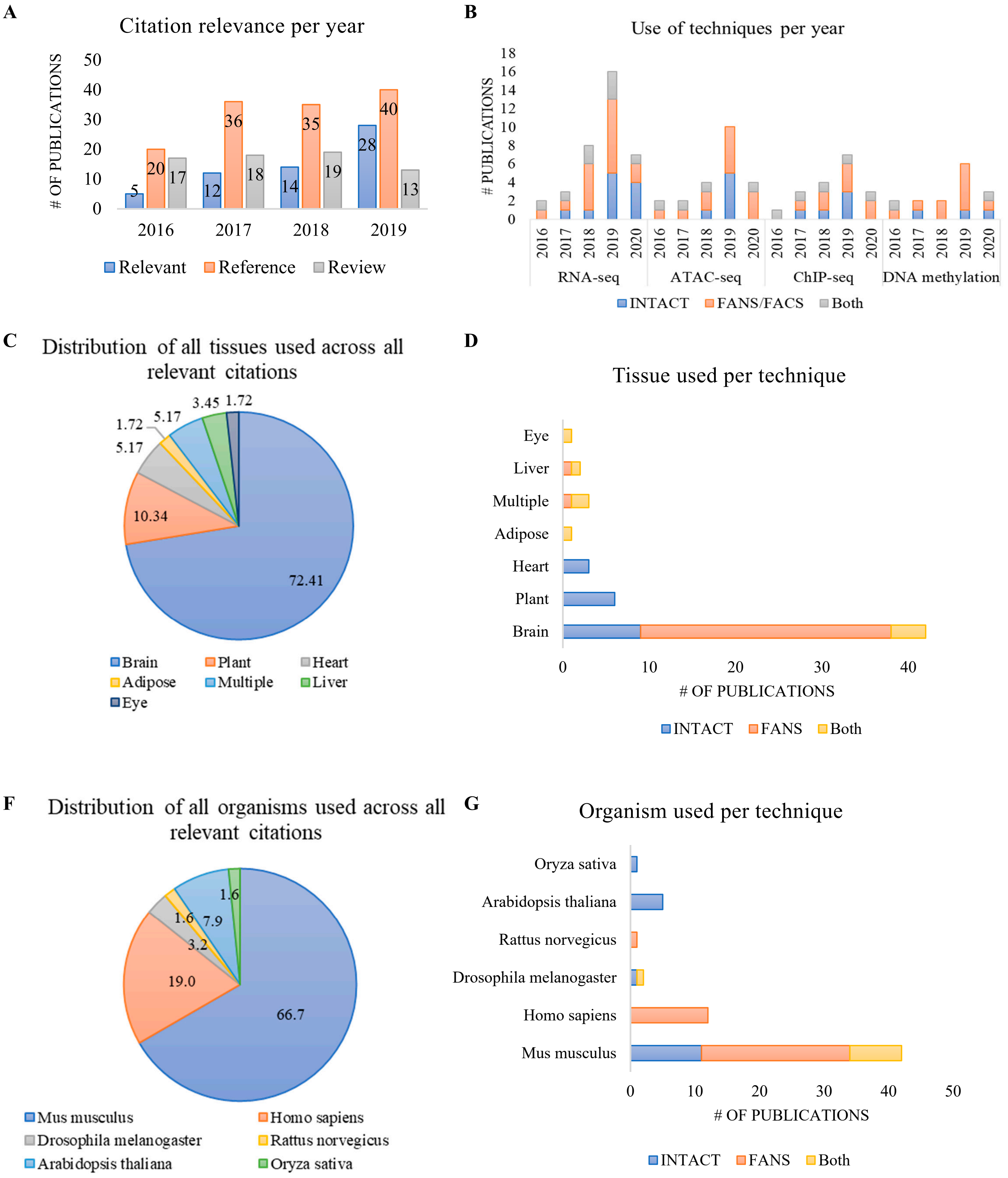
A
Citation relevance per year
# OF PUBLICATIONS
50
40
30
20
10
0
5
20
17
12
36
18
14
35
19
28
40
13
2016
2017
2018
2019
Relevant
Reference
Review
B
Use of techniques per year
# PUBLICATIONS
18
16
14
12
10
8
6
4
2
0
2016
2017
2018
2019
2020
RNA-seq
ATAC-seq
ChIP-seq
DNA methylation
INTACT
FANS/FACS
Both
C
Distribution of all tissues used across all relevant citations
3.45
1.72
1.72
5.17
5.17
10.34
72.41
Brain
Plant
Heart
Adipose
Multiple
Liver
Eye
D
Tissue used per technique
Eye
Liver
Multiple
Adipose
Heart
Plant
Brain
0
10
20
30
40
# OF PUBLICATIONS
INTACT
FANS
Both
F
Distribution of all organisms used across all relevant citations
1.6
1.6
7.9
3.2
19.0
66.7
Mus musculus
Homo sapiens
Drosophila melanogaster
Rattus norvegicus
Arabidopsis thaliana
Oryza sativa
G
Organism used per technique
Oryza sativa
Arabidopsis thaliana
Rattus norvegicus
Drosophila melanogaster
Homo sapiens
Mus musculus
0
10
20
30
40
50
# OF PUBLICATIONS
INTACT
FANS
Both

**Supplementary Figure S1.** Literature review of Mo et al. (2015) citations. **A.** Classification of "relevant", and "non-relevant" ("reference" and "review") publications per year. **B.** Number of publications using the selected techniques per year. **C.** Distribution of all tissues across all relevant publications irrespective of technique. **D.** Classification of tissue used per technique **E.** Distribution of all organism across all relevant publications irrespective of technique. **F.** Classification of organisms used per technique.

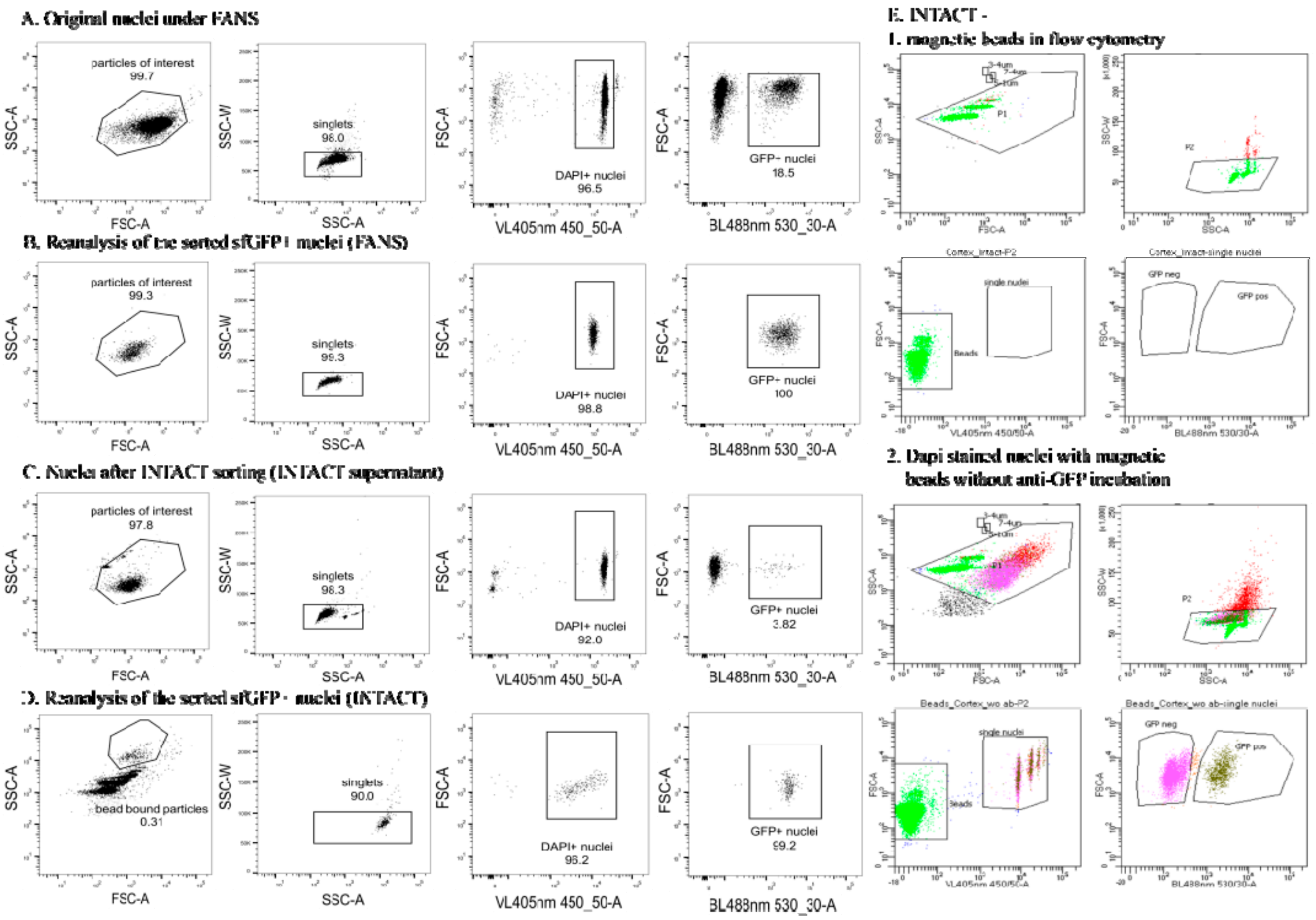


**Supplementary Figure S2.** Steps leading to the purity analysis of sorted nuclei using flow cytometry. **A.** Input nuclei undergoing FANS sorting. **B.** Reanalysis of the FANS sorted sfGFP+ nuclei. **C.** Analysis of supernatant remaining after INTACT bead purification. **D.** Reanalysis of the INTACT sorted sfGFP+ nuclei (anti-GFP incubated nuclei). **E.** Establishing flow cytometry sorting for samples with magnetic beads.

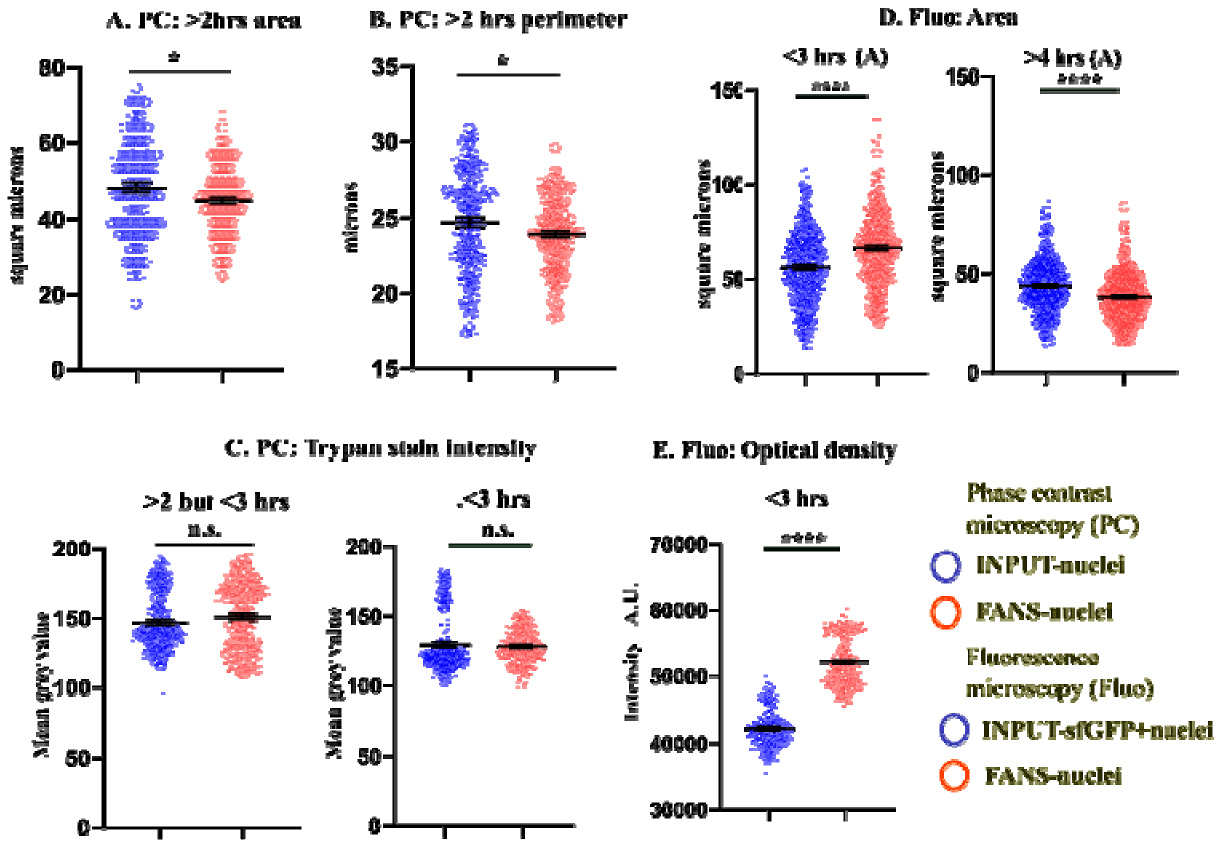


**Supplementary Figure S3.** Additional parameter changes in FANS nuclei as compared to INPUT nuclei at different time points after sorting PC: Phase Contrast Microscopy, Fluo: Fluorescence Microscopy **A.** Area of nuclei observed within 2 hours ($p < 0.05$) **B.** Perimeter of nuclei observed within 2 hours ($p < 0.05$) **C.** Trypan staining intensity observed within 3 hours (n.s., n.s.) **D.** Automated area calculations for nuclei embedded in the IBIDI chamber at different time points after the sorting ($p < 0.0001$, $p < 0.0001$) **E.** Optical density of sorted nuclei ($p < 0.0001$)

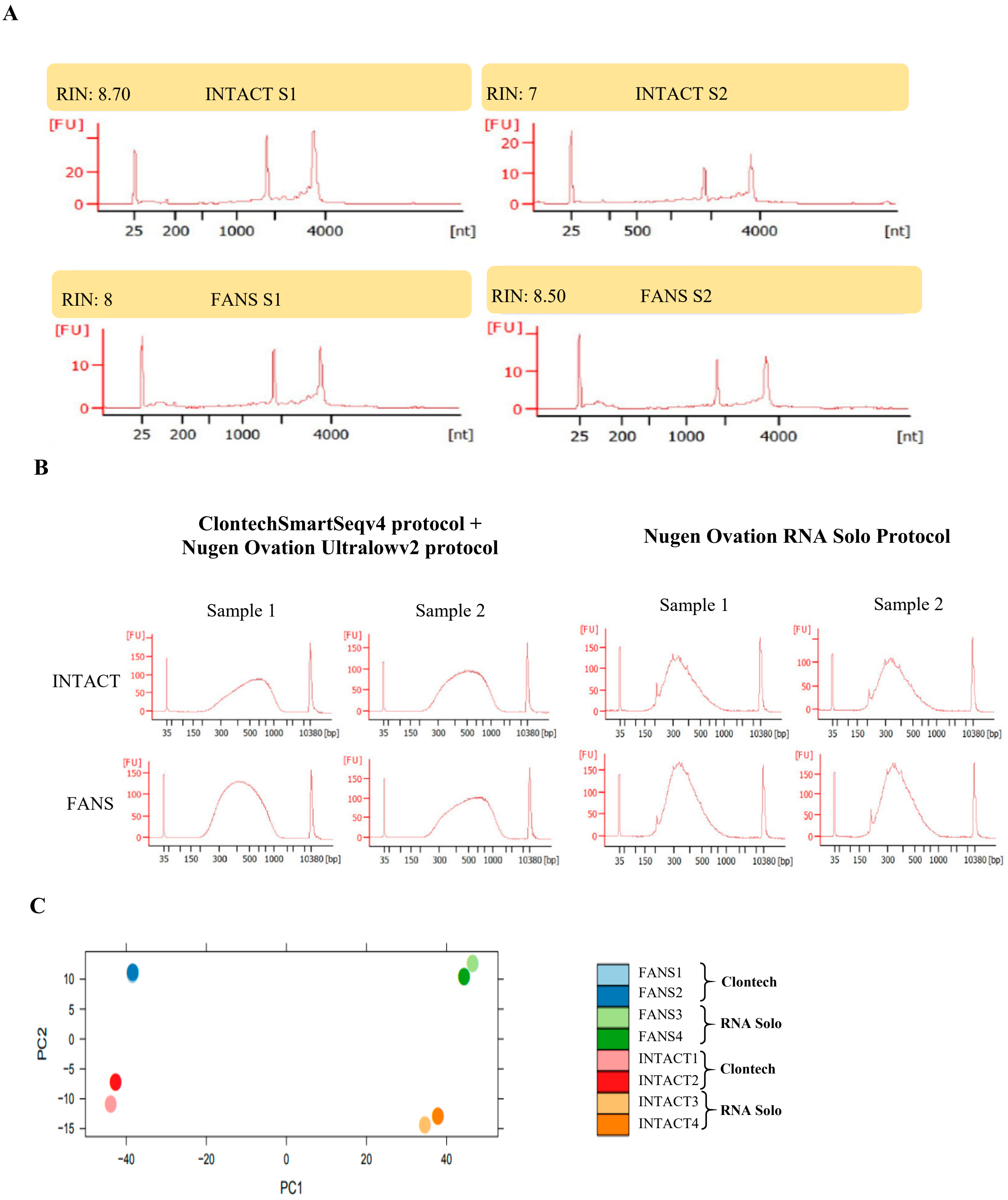


**Supplementary Figure S4.** Selection of RNA library preparation strategy**. A.** Bioanalyzer profile of hippocampus nuclei RNA (*n*= 2 INTACT and *n*= 2 FANS *per* library preparation). **B.** Bioanalyzer profile of amplified libraries using either Clontech Smartseq v4 protocol (Clontech)

in combination with Nugen Ovation ultralow2 (Nugen) or Nugen ovation RNA solo protocol. **C.** PCA clustering analysis of FANS and INTACT samples based on library preparation.

**A**

| Group 1 | | Group 2 | | Group 3 | |
|---|---|---|---|---|---|
| **Sample** | **RIN** | **Sample** | **RIN** | **Sample** | **RIN** |
| FANS 1 | 9.9 | FANS 1 | 4.2 | FANS 1 | 5.1 |
| FANS 2 | 9.9 | FANS 2 | 4.0 | FANS 2 | 5.7 |
| FANS 3 | 9.7 | FANS 3 | 6.1 | FANS 3 | 4.1 |
| FANS 4 | 8.0 | FANS 4 | 5.9 | FANS 4 | 5.2 |
| INTACT 1 | 7.6 | FANS 5 | 4.9 | FANS 5 | 5.3 |
| INTACT 2 | 8.6 | FANS 6 | 5.9 | FANS 6 | 4.9 |
| INTACT 3 | 7.4 | FANS 7 | 6.6 | FANS 7 | 6.1 |
| INTACT 4 | 6.3 | INTACT 1 | 7.5 | INTACT 1 | 4.4 |
| | | INTACT 2 | 7 | INTACT 2 | 5.6 |
| | | INTACT 3 | 5.9 | INTACT 3 | 6.1 |
| | | INTACT 4 | 5.2 | INTACT 4 | 6.4 |
| | | INTACT 5 | 6.7 | INTACT 5 | 5.8 |
| **RIN Range: 6.3 - 9.9** | | **RIN Range: 4.0 - 7.5** | | **RIN Range: 4.1 - 6.4** | |

**B**

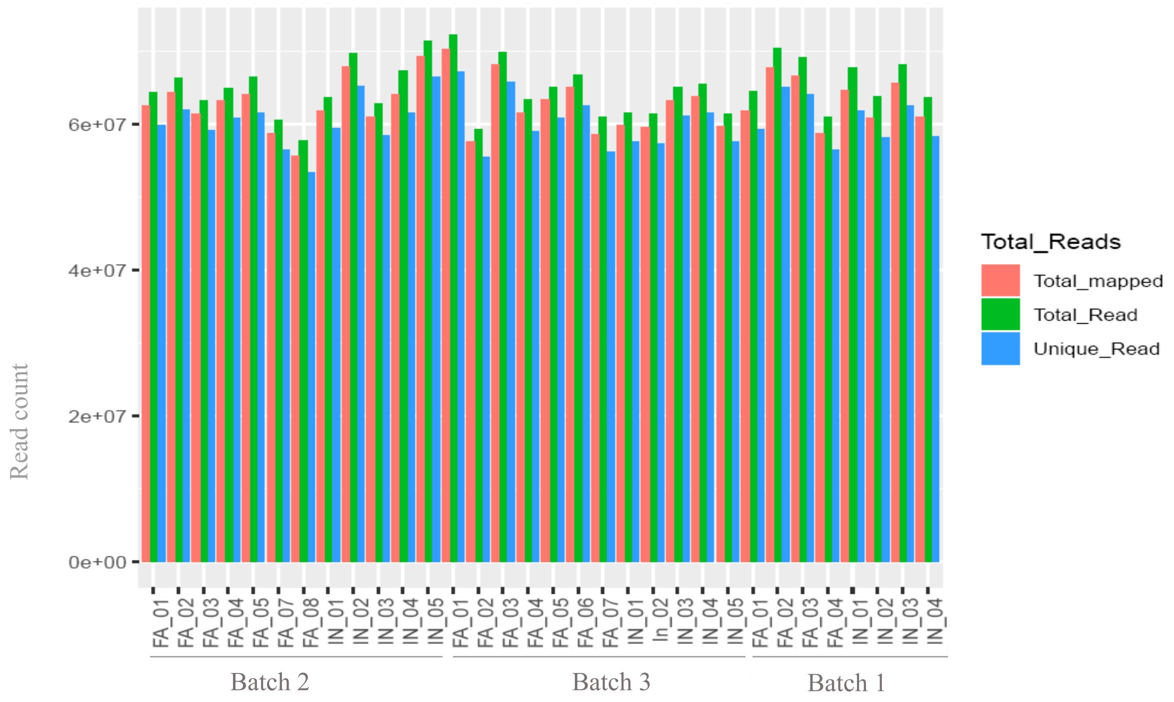


**Supplementary Figure S5.** Sequencing statistics of three batches with distinct RIN values. **A.** Individual RIN values of the sequenced samples and the total range of RIN values. **B.** Read count all of the samples including total reads, mapped reads and unique reads.

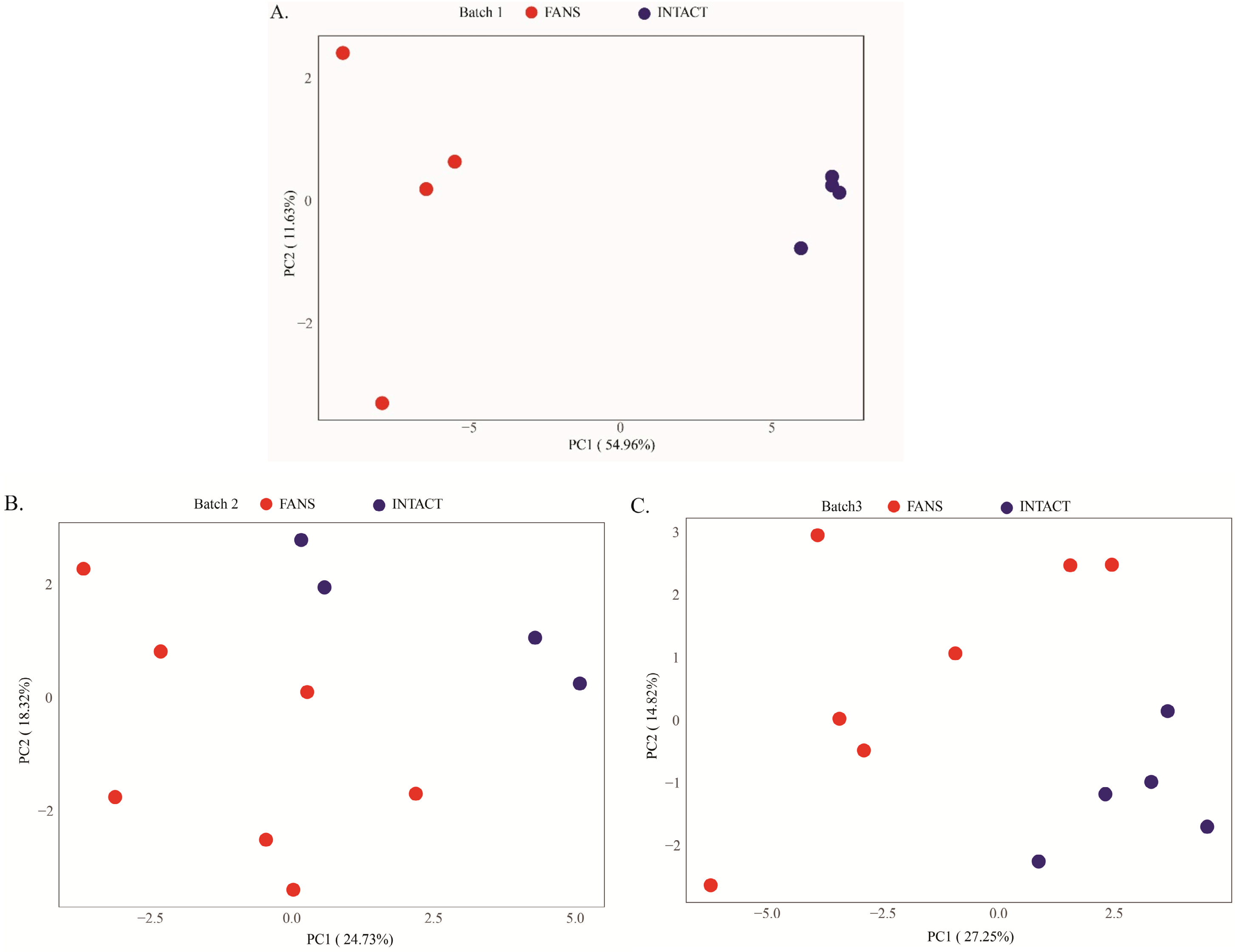


**Supplementary Figure S6.** Target gene and clustering comparison between the distinct batch samples. **A–C** Batch-specific PCA comparison of batch 1 (**A**), batch 2 (**B**) and batch 3 (**C**).

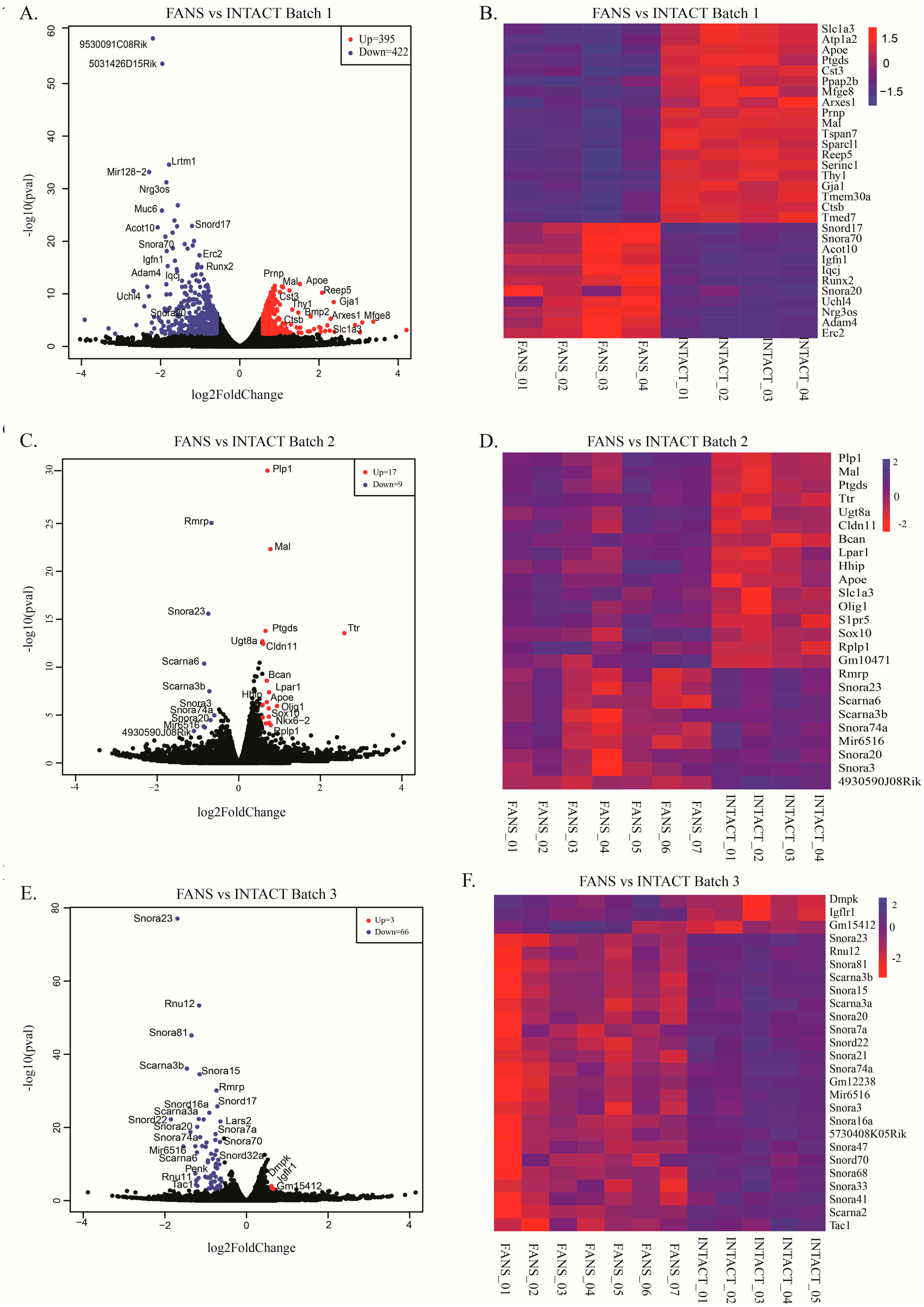
A.
FANS vs INTACT Batch 1
Up=395
Down=422
9530091C08Rik
5031426D15Rik
Lrtm1
Mir128−2
Nrg3os
Muc6
Snord17
Acot10
Snora70
Erc2
Igfn1
Runx2
Adam4
Iqcj
Uchl4
Snora20
Prnp
Mal
Apoe
Reep5
Cst3
Gja1
Thy1
Bmp2
Arxes1
Mfge8
Ctsb
Slc1a3
-log10(pval)
log2FoldChange
B.
FANS vs INTACT Batch 1
Slc1a3
Atp1a2
Apoe
Ptgds
Cst3
Ppap2b
Mfge8
Arxes1
Prnp
Mal
Tspan7
Sparcl1
Reep5
Serinc1
Thy1
Gja1
Tmem30a
Ctsb
Tmed7
Snord17
Snora70
Acot10
Igfn1
Iqcj
Runx2
Snora20
Uchl4
Nrg3os
Adam4
Erc2
1.5
0
−1.5
FANS_01
FANS_02
FANS_03
FANS_04
INTACT_01
INTACT_02
INTACT_03
INTACT_04
C.
FANS vs INTACT Batch 2
Up=17
Down=9
Plp1
Rmrp
Mal
Snora23
Ptgds
Ttr
Ugt8a
Cldn11
Scarna6
Bcan
Scarna3b
Lpar1
Hhip
Apoe
Snora3
Olig1
Snora74a
Sox10
Snora20
Nkx6−2
Mir6516
Rplp1
4930590J08Rik
-log10(pval)
log2FoldChange
D.
FANS vs INTACT Batch 2
Plp1
Mal
Ptgds
Ttr
Ugt8a
Cldn11
Bcan
Lpar1
Hhip
Apoe
Slc1a3
Olig1
S1pr5
Sox10
Rplp1
Gm10471
Rmrp
Snora23
Scarna6
Scarna3b
Snora74a
Mir6516
Snora20
Snora3
4930590J08Rik
2
0
-2
FANS_01
FANS_02
FANS_03
FANS_04
FANS_05
FANS_06
FANS_07
INTACT_01
INTACT_02
INTACT_03
INTACT_04
E.
FANS vs INTACT Batch 3
Up=3
Down=66
Snora23
Rnu12
Snora81
Scarna3b
Snora15
Rmrp
Snord16a
Snord17
Scarna3a
Snord22
Lars2
Snora20
Snora7a
Snora74a
Snora70
Mir6516
Snord32a
Scarna6
Dmpk
Penk
Igflr1
Rnu11
Tac1
Gm15412
-log10(pval)
log2FoldChange
F.
FANS vs INTACT Batch 3
Dmpk
Igflr1
Gm15412
Snora23
Rnu12
Snora81
Scarna3b
Snora15
Scarna3a
Snora20
Snora7a
Snord22
Snora21
Snora74a
Gm12238
Mir6516
Snora3
Snora16a
5730408K05Rik
Snora47
Snord70
Snora68
Snora33
Snora41
Scarna2
Tac1
2
0
-2
FANS_01
FANS_02
FANS_03
FANS_04
FANS_05
FANS_06
FANS_07
INTACT_01
INTACT_02
INTACT_03
INTACT_04
INTACT_05

**Supplementary Figure S7.** Differentially expressed genes (DEGs) between INTACT and FANS. **A–F.** Volcano Plot and heat-map representing the DEGs of INTACT compared to FANS in batch 1 (**A,B**), batch 2 (**C,D**) and batch 3 (**E,F**). Only Snora20 was depleted INTACT compared to FANS, in all batches. (All batches represent 1.5 fold change $p < 0.05$).

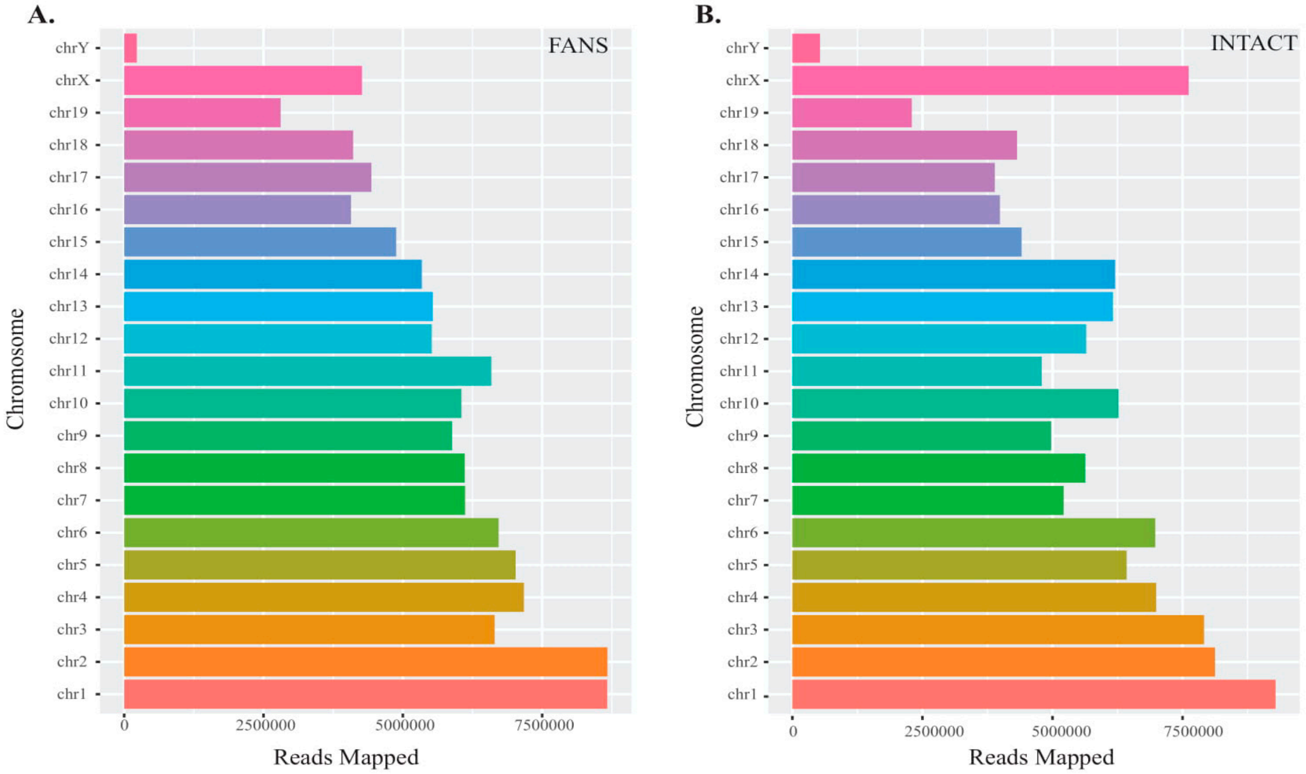


**Supplementary Figure S8.** FANS and INTACT ATAC-Seq reads mapped per chromosome.

**A.** Gained accessibility peak motifs

| Motif | TF | P-value | Fraction |
|---|---|---|---|
| 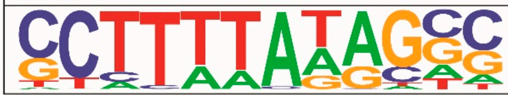 | TATA-Box(TBP) | 1e-2 | 30.00 % |

**B.** Reduced accessibility peak motifs

| Motif | TF | P-value | Fraction |
|---|---|---|---|
| 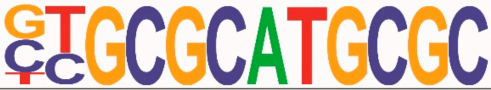 | NRF | 1e-10 | 21.10 % |
| 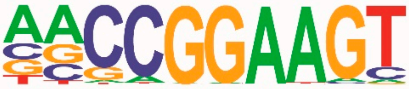 | ETS | 1e-9 | 24.24 % |
| 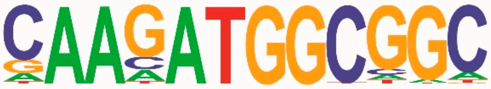 | YY1 | 1e-8 | 7.23 % |
| 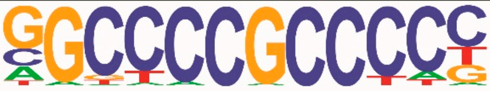 | SP1 | 1e-7 | 41.43 % |
| 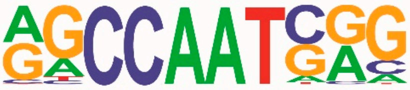 | NFY | 1e-5 | 29.56 % |
| 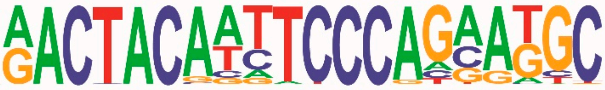 | GFY- Staf | 1e-2 | 6.37 % |

**Supplementary Figure S9.** Motif enrichment analysis at promoters regions of INTACT-gained accessibility (**A**) and INTACT-reduced accessibility (**B**) peak motifs.
